# Integration of Bioinformatics and Machine Learning Strategies Identifies Ferroptosis and Immune Infiltration Signatures in Peri-Implantitis

**DOI:** 10.3390/ijms26094306

**Published:** 2025-05-01

**Authors:** Jieying Huang, Yaokun Zou, Huizhi Deng, Jun Zha, Janak Lal Pathak, Yaxin Chen, Qing Ge, Liping Wang

**Affiliations:** Department of Oral Implantology, School and Hospital of Stomatology, Guangdong Engineering Research Center of Oral Restoration and Reconstruction & Guangzhou Key Laboratory of Basic and Applied Research of Oral Regenerative Medicine, Guangzhou Medical University, Guangzhou 510182, China; huangjieying@stu.gzhmu.edu.cn (J.H.); wilsonzaw000@stu.gzhmu.edu.cn (Y.Z.)

**Keywords:** peri-implantitis, ferroptosis, FLT3, TLR4, machine learning, immune infiltration

## Abstract

Peri-implantitis (PI) is a chronic inflammatory disease that ultimately leads to the dysfunction and loss of implants with established osseointegration. Ferroptosis has been implicated in the progression of PI, but its precise mechanisms remain unclear. This study explores the molecular mechanisms of ferroptosis in the pathology of PI through bioinformatics, offering new insights into its diagnosis and treatment. The microarray datasets for PI (GSE33774 and GSE106090) were retrieved from the GEO database. The differentially expressed genes (DEGs) and ferroptosis-related genes (FRGs) were intersected to obtain PI-Ferr-DEGs. Using three machine learning algorithms, the Least Absolute Shrinkage and Selection Operator (LASSO), Support Vector Machine-Recursive Feature Elimination (SVM-RFE), and Boruta, we successfully identified the most crucial biomarkers. Additionally, these key biomarkers were validated using a verification dataset (GSE223924). Gene set enrichment analysis (GSEA) was also utilized to analyze the associated gene enrichment pathways. Moreover, immune cell infiltration analysis compared the differential immune cell profiles between PI and control samples. Also, we targeted biomarkers for drug prediction and conducted molecular docking analysis on drugs with potential development value. A total of 13 PI-Ferr-DEGs were recognized. Machine learning and validation confirmed toll-like receptor-4 (TLR4) and FMS-like tyrosine kinase 3 (FLT3) as ferroptosis biomarkers in PI. In addition, GSEA was significantly enriched by the biomarkers in the cytokine–cytokine receptor interaction and chemokine signaling pathway. Immune infiltration analysis revealed that the levels of B cells, M1 macrophages, and natural killer cells differed significantly in PI. Ibudilast and fedratinib were predicted as potential drugs for PI that target TLR4 and FLT3, respectively. Finally, the occurrence of ferroptosis and the expression of the identified key markers in gingival fibroblasts under inflammatory conditions were validated by RT-qPCR and immunofluorescence analysis. This study identified TLR4 and FLT3 as ferroptosis and immune cell infiltration signatures in PI, unraveling potential novel targets to treat PI.

## 1. Introduction

Peri-implantitis (PI), first defined at the European Workshop on Periodontology in 1993, refers to the inflammatory process affecting the soft and hard tissue surrounding a functioning dental implant that has achieved osseointegration. Clinically, PI presents with signs such as redness and swelling of the PI mucosa, bleeding on probing, increased probing depth, and radiographic evidence of vertical bone loss around the implant [1]. It has been reported that the prevalence of PI can be as high as 56% [2]. With the development of implant technology and the expansion of treatment indications, more and more patients with dentition defects or complete edentulism choose implant treatment [3]. Consequently, the prevention and management of PI pose a formidable challenge in oral implantology. The etiology of PI is multifactorial, involving a complex interplay of various risk factors such as a history of periodontitis [4], diabetes [5], smoking [6], residual cement [7], and plaque biofilm [8]. The early diagnosis of PI is often challenging, leading to delays in treatment that exacerbate the condition. Additionally, compared to natural teeth, the structural characteristics of PI gingival collagen fibers, their relatively limited blood supply, and differences in the proportion of immune cells make the PI tissues inherently a biological barrier with lower defense capabilities [9,10,11]. As a result, inflammation tends to progress more rapidly in PI. Therefore, it is prudent to investigate the mechanisms underlying the onset and progression of PI to establish a foundation for its early diagnosis and treatment.

Ferroptosis is an iron-dependent form of regulated cell death characterized by lipid peroxidation accumulation and the inactivation of glutathione peroxidase 4 (GPX4) [12]. Recent studies suggest that ferroptosis may play an essential role in the development of PI. Using single-cell analysis, Li J et al. found that ferroptosis in the PI group was significantly more severe than that in the healthy group and periodontitis group, and Acyl-CoA Synthetase Long-Chain Family Member 4 (ACSL4), one of the key genes regulating ferroptosis, was significantly expressed [13]. Another study found that blocking ferroptosis could alleviate PI-induced osteogenic inhibition of bone-marrow-derived mesenchymal stem cells (BMSCs) [14]. As we know, reactive oxygen species (ROS), generated by iron-catalyzed reactions, are key factors driving lipid peroxidation and ultimately leading to cell ferroptosis. Recently, a study pointed out that the oxidative DNA damage marker 8-hydroxydeoxyguanosine (8-OHdG) and lipid peroxidation product malondialdehyde (MDA) in saliva derived from PI patients were significantly higher than those in the healthy control group and periodontitis group. At the same time, the antioxidant enzyme glutathione peroxidase (GPX) was reduced considerably, and superoxide dismutase (SOD) was significantly increased [15]. The differential expression of these markers suggests an imbalance in the overall antioxidant system during the pathological process of PI, especially the imbalance between the regulation of ROS production and clearance, which may be related to the regulation of ferroptosis and inflammatory response. Other researchers also observed similar results in the gingival crevicular fluid of PI patients [16]. In PI, periodontal tissue cells can undergo ferroptosis due to this high oxidative stress state, causing cell death and further tissue destruction, eventually leading to the failure of implant survival. Therefore, an in-depth study of the relationship between ferroptosis and PI may provide a new perspective for developing new diagnostic and therapeutic strategies to improve the long-term stability of implants and patients’ quality of life.

Overall, existing research suggests that the occurrence and development of PI are closely associated with ferroptosis caused by oxidative stress in its microenvironment. However, no studies have yet delved deeply into the role of ferroptosis in this process. Machine learning has become a powerful tool in biomedical research, particularly for identifying potential biomarkers [17,18]. The Least Absolute Shrinkage and Selection Operator (LASSO) is highly effective in feature selection by reducing overfitting due to its penalization of significant coefficients [19]. Support Vector Machine-Recursive Feature Elimination (SVM-RFE) excels through its iterative process of ranking feature importance, leading to the identification of optimal biomarker subsets [20]. Boruta, a wrapper algorithm built around random forests, is particularly robust in identifying all relevant features by comparing their importance to shuffled variables [21]. In this study, bioinformatics analysis and the above three machine learning algorithms were integrated to identify the ferroptosis-related differentially expressed genes (PI-Ferr-DEGs) with diagnostic value in PI, to explore their potential mechanism in the pathological process of PI and to screen out promising therapeutic drugs.

In addition, PI involves complex interactions among various types of cells. Single-cell analysis has revealed a significant increase in the accumulation of type 2 fibroblasts within PI, which exhibit a pro-inflammatory phenotype and heightened immune response [22]. These findings suggest that the immune microenvironment of PI warrants particular attention. In our study, we performed immune infiltration analysis on PI and healthy tissue samples and explored the relationship between the identified diagnostic markers and immune infiltrating cells. This study identified toll-like receptor-4 (TLR4) and FMS-like tyrosine kinase 3 (FLT3) as ferroptosis and immune cell infiltration signatures in PI, unraveling potential novel targets to treat PI. Numerous studies have confirmed that LPS, as a key virulence factor of oral pathogens such as *Porphyromonas gingivalis*, can induce pro-inflammatory phenotypes in HGFs, making it a well-established experimental tool for constructing inflammation-related in vitro models [14,23,24,25,26]. Single-cell sequencing has further revealed a significant reduction in fibroblast populations and elevated proportions of CXCL13^+^ pro-inflammatory subsets within PI lesions [13]. Therefore, investigating ferroptosis dynamics and the expression of key biomarkers in this inflammatory state model of HGFs provides valuable preliminary validation for bioinformatics predictions. Finally, we established an inflammatory state model of HGFs by adding LPS to the culture medium and we performed immunofluorescence staining and RT-qPCR analysis on the samples [27,28]. The results showed that TLR4 expression was upregulated, while GPX4/Solute Carrier Family 7 Member 11 (SLC7A11) expression in the classical ferroptosis pathway was downregulated. The downregulation of GPX4/SLC7A11 may reflect the occurrence of oxidative stress and ferroptosis, suggesting that this process could play a crucial role in the damage and inflammatory response of gingival fibroblasts.

## 2. Results

### 2.1. Data Collection and Preprocessing

**Figure 1:**
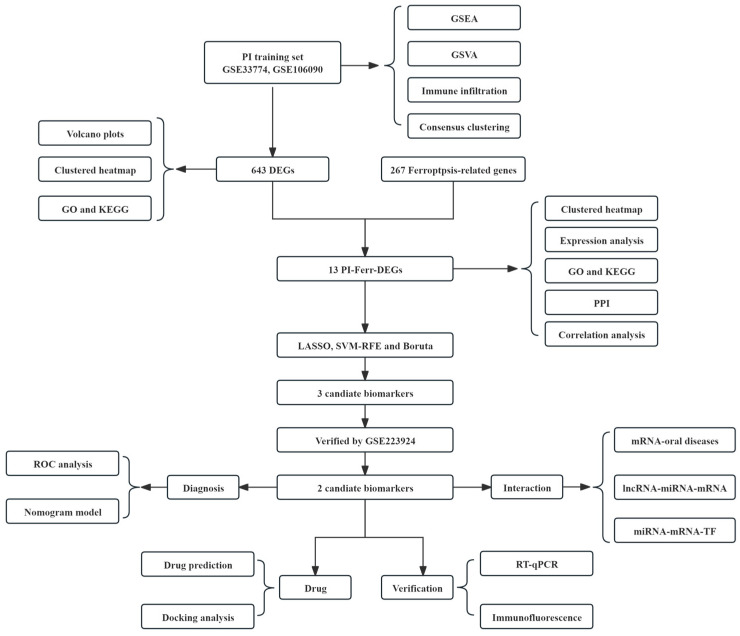
Design flow chart.

Figure 1 illustrates the overall study design process. To increase the signal and reduce the proportion of false-positive results, GSE106090 and GSE33774 were normalized in a batch to minimize variability. Before normalization, the median and quartiles of the sample data in the training set differed significantly in some samples. The median of GSM835234–GSM835248 was lower, while that of GSM2829420 and subsequent samples was higher with more outliers. After normalization, the data distribution was highly consistent across the median and quartiles among all samples, indicating more reliable and consistent corrected data (Supplemental Figure S1A). Data density plots demonstrated the corrected, more uniform density distribution of gene expression, eliminating the bias between the original datasets (Supplemental Figure S1B). Furthermore, in the PCA plots, the distributions of the two datasets exhibited significant overlap. This suggests that the employed Combat method made the distributions of the two datasets within the same feature space more consistent, thereby reducing the systematic differences between them (Supplemental Figure S1C). Similarly, the control and disease groups enhanced the biological relevance of the data, making the differences between the groups more realistic and convenient for subsequent analyses (Supplemental Figure S1D).

### 2.2. Identification and Functional Analysis of DEGs

**Figure 2:**
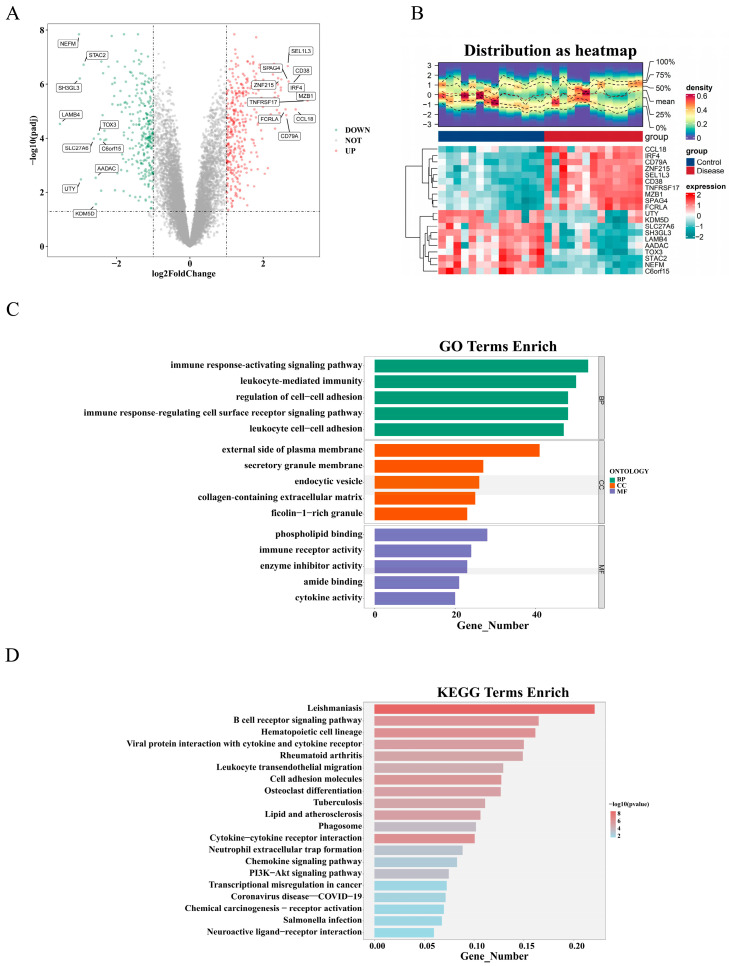
Identification and functional analysis of DEGs. (**A**) Volcano plots; the up-expressed mRNAs are shown in red. The down-expressed mRNAs are shown in green,, and the top 10 significantly up-regulated and down-regulated genes are marked, respectively, in the figure (Difference analysis threshold: |log2FC| ≥ 1, adj. *p* < 0.05). (**B**) Clustered heatmap of DEGs. (**C**) Enriched items in GO terms analysis of DEGs. (**D**) Enriched pathways in KEGG analysis of DEGs. DEGs, differentially expressed genes; GO, Gene Ontology; BP, biological process; CC, cellular component; MF, molecular function; KEGG, Kyoto Encyclopedia of Genes and Genomes.

Through differential analysis in the training set, 643 DEGs were identified. Differential expression analysis demonstrated that 374 genes were significantly up-regulated, whereas 269 genes were down-regulated in the disease group. Furthermore, the volcano plot and heat map displayed the top 10 (up/down) regulated genes (Figure 2A,B). Subsequently, GO enrichment analysis was conducted on the 643 DEGs to elucidate the molecular biological processes differentiating the PI group from the control group. A total of 1317 GO biological functions were enriched, and the results indicated that DEGs had an immune response-activating signaling pathway, leukocyte-mediated immunity, and regulation of cell–cell adhesion were significantly enriched in 1107 GO-BPs; in 62 GO-CCs, there was the external side of the plasma membrane and endocytic vesicle. And 148 GO-MFs had significant enrichment in phospholipid binding, immune receptor activity, and enzyme inhibitor activity (Figure 2C). The DEGs were also enriched for 64 KEGG pathways, with the top 20 most significantly enriched pathways including the cytokine–cytokine receptor interaction, the PI3K-Akt signaling pathway, lipid and atherosclerosis, and cell adhesion molecules (Figure 2D).

### 2.3. Comprehensive Functional Analysis of PI-Ferr-DEGs

**Figure 3:**
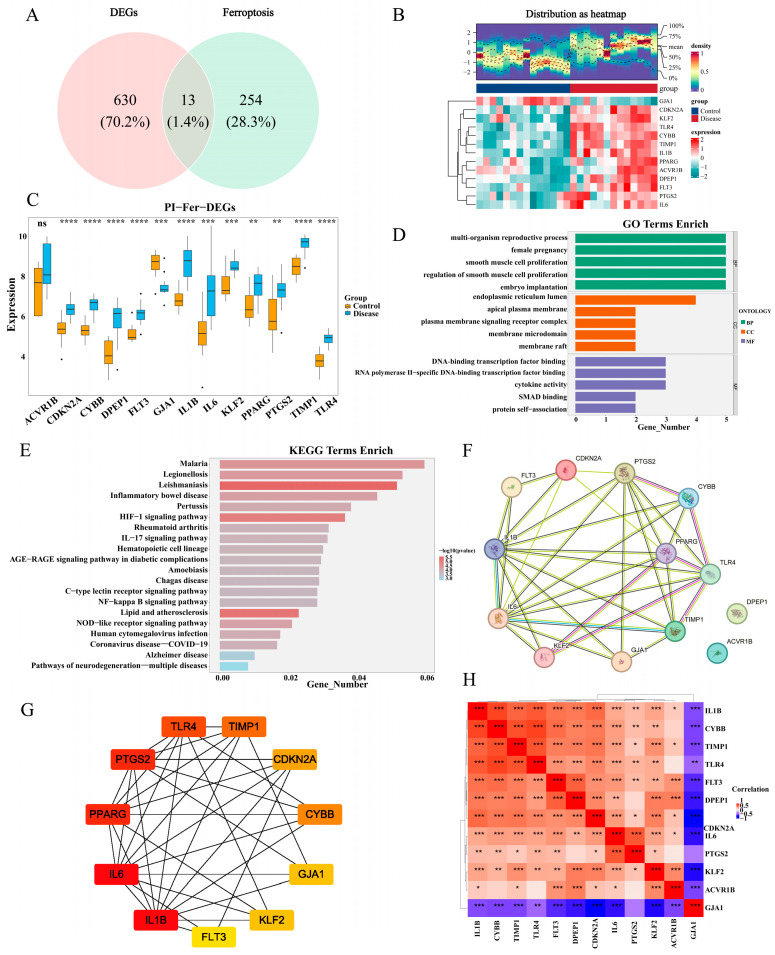
Comprehensive functional analysis of PI-Ferr-DEGs. (**A**) Venn diagram, determination of 13 PI-Ferr-DEGs. (**B**) PI-Ferr-DEGs heat map and density distribution map. (**C**) The box plot shows the mRNA expression of 13 PI-Ferr-DEGs between the control group and disease group (**D**) Enriched items in GO terms analysis of PI-Ferr-DEGs. (**E**) Enriched pathways in KEGG analysis of PI-Ferr-DEGs. (**F**,**G**) PPI network analysis of PI-Ferr-DEGs. (**H**) In the correlation analysis of PI-Ferr-DEGs, red represents a positive correlation, and blue represents a negative correlation. PI, peri-implantitis; Ferr, ferroptosis; DEGs, differentially expressed genes; GO, Gene Ontology; BP, biological process; CC, cellular component; MF, molecular function; KEGG, Kyoto Encyclopedia of Genes and Genomes; PPI, protein–protein interaction. Ns: not significant; * *p* < 0.05; ** *p* < 0.01; *** *p* < 0.001; **** *p* < 0.0001.

The intersection between 643 DEGs and 267 FRGs was taken, and the result was illustrated using a Venn diagram (Figure 3A). In total, 13 PI-Ferr-DEGs were obtained for subsequent analyses, namely, CYBB, FLT3, CDKN2A, GJA1, TLR4, IL1B, IL6, PPARG, KLF2, DPEP1, GJA1, TIMP1, and PTGS2. Except for ACVR1B, whose up-regulation was not significant in the disease group, the remaining PI-Ferr-DEGs exhibited statistically significant differences between both the disease and control groups (*p* < 0.05) (Figure 3B). The heatmap and density profile of gene expression in PI-Ferr-DEGs visually demonstrated gene expression differences between the two groups (Figure 3C).

In addition, GO and KEGG enrichment analyses of PI-Ferr-DEGs were analyzed to reveal the underlying molecular biological processes. The results of 1076 GO enrichment were obtained, and 984 BP fractions were mainly enriched in processes such as multi-organism reproductive process, female pregnancy, and smooth muscle cell proliferation. A total of 21 CC fractions were primarily enriched in endoplasmic reticulum lumen, apical plasma membrane, and plasma membrane signaling receptor complex. A total of 71 MF fractions were mainly enriched in functions such as DNA-binding transcription factor binding and RNA polymerase II-specific DNA-binding transcription factor binding (Figure 3D), and the 47 KEGG pathways were primarily enriched in lipid and atherosclerosis, leishmaniasis, the HIF-1 signaling pathway, and the NOD-like receptor signaling pathway (Figure 3E).

Following this, the protein interactions among the PI-Ferr-DEGs were investigated, resulting in the protein interaction network comprising 13 nodes and 35 interaction pairs (confidence > 0.4). Within this network, TLR4 and PPARG demonstrated extensive interactions with other genes and might play significant regulatory roles in this network (Figure 3F). Subsequently, the NCC method indicated that genes like IL1B, IL6, and PPARG were recognized as key nodes in the PPI network (Figure 3G). Moreover, IL1B and IL6 are inflammation-related factors and are commonly expressed in the inflammatory response, exhibiting a strong positive correlation. TLR4 was also a positively associated gene pair with CYBB and IL1B. They act as key factors in the immune response, and the high correlations among these genes reflect their typical role in the immune system [29]. In contrast, GJA1 negatively correlated with IL6 and IL1B, suggesting they may play opposing roles in different biological pathways (Figure 3H). These analyses offered perspectives on the molecular mechanisms underlying PI and highlighted promising pathways of PI for therapeutic targeting.

### 2.4. The Identification and Validation of TLR4 and FLT3

**Figure 4:**
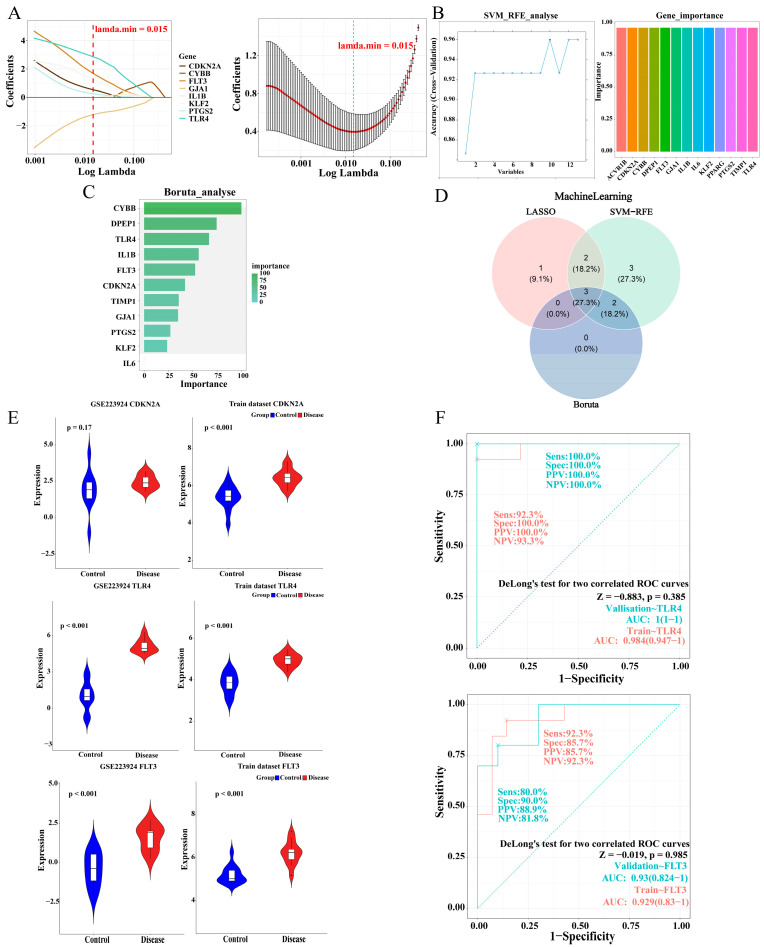
Machine learning screening to obtain candidate biomarkers and their verification. (**A**) LASSO regression analysis of PI-Ferr-DEGs. (**B**) SVM-RFE cross-validation accuracy and gene importance of PI-Ferr-DEGs. (**C**) Boruta analysis of PI-Ferr-DEGs. (**D**) The Venn diagram shows the candidate markers identified by the three machine learning methods. (**E**) The violin plot showed the difference of candidate biomarkers between the validation set and the training set. (**F**) The ROC curves, respectively, demonstrate the classification ability of TLR4 and FLT3 as biomarkers between the disease and control groups. PI, peri-implantitis; Ferr, ferroptosis; DEGs, differentially expressed genes.

Models were constructed using the LASSO, SVM-RFE, and Boruta algorithms to identify feature genes associated with PI and FRGs. First, LASSO analysis yielded six feature genes, which were FLT3, CDKN2A, TLR4, KLF2, GJA1, and PTGS2 (lambda. min = 0.015) (Figure 4A). In SVM-RFE analysis, the model prediction accuracy was highest with 10 feature genes, which included CYBB, TLR4, IL1B, CDKN2A, FLT3, TIMP1, DPEP1, IL6, GJA1, and KLF2 (Figure 4B). Subsequent Boruta analysis identified all five feature genes with importance as confirmed, including CYBB, DPEP1, TLR4, IL1B, and FLT3 (Figure 4C). The above three machine learning algorithms were screened to cross the feature genes of PI associated with FRGs, resulting in three candidate biomarkers, namely FLT3, TLR4, and CDKN2A (Figure 4D). After validating the expression trends of the three candidate biomarkers in both the disease and control groups in the training set and validation set, it was found that TLR4 and FLT3 were up-regulated, exhibiting significant expression in the disease group (*p* < 0.05) (Figure 4E). Next, the outcomes of ROC validation demonstrated that the AUCs of TLR4 and FLT3 were more significant than 0.9 in both datasets (Figure 4F). This further enhanced the accuracy of TLR4 and FLT3 as biomarkers.

### 2.5. Construction and Assessment of a Nomogram for PI Diagnosis

**Figure 5:**
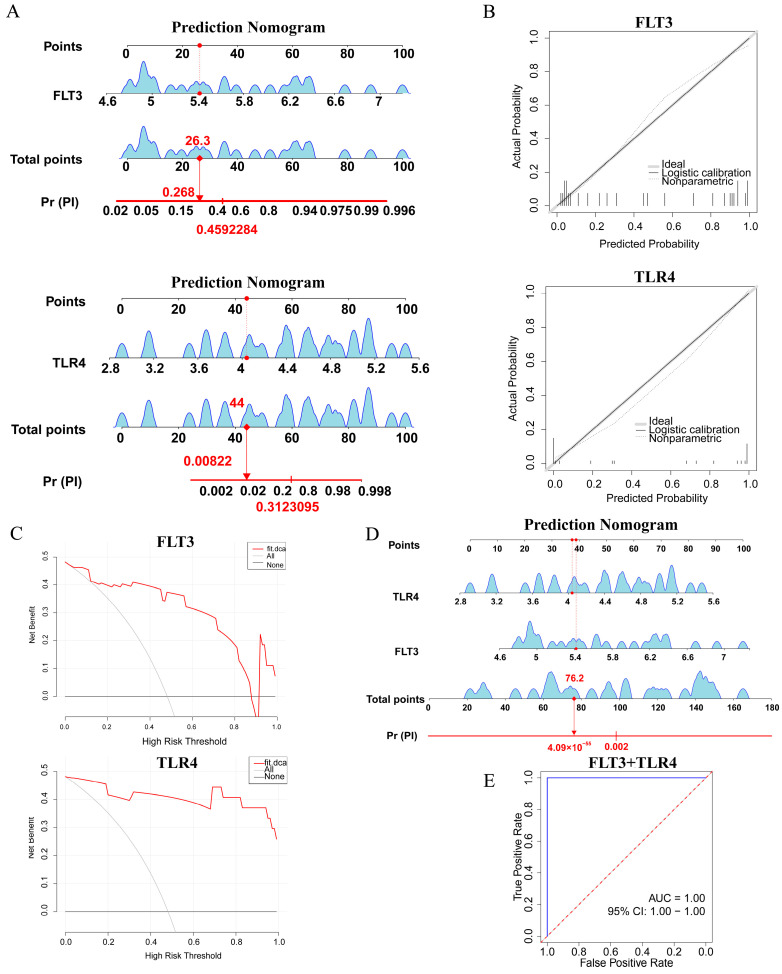
The diagnostic performance of FLT3 and TLR4 for predicting PI was evaluated. (**A**) Nomogram. (**B**) Calibration curve. (**C**) Decision Curve Analysis. (**D**) Nomogram of biomarkers combination. (**E**) The ROC curves of biomarkers combination. ROC, Receiver Operating Characteristic; AUC, Area Under the Curve.

A nomogram model was developed for PI diagnosis based on TLR4 and FLT3 in the training set (Figure 5A). Subsequently, the calibration plot illustrated a close alignment between the calibration prediction curve and the ideal curve, suggesting an excellent agreement between the actual and predicted probabilities of PI diagnosis (Figure 5B). The nomogram generated from two biomarkers showed that the red curve shows a higher net gain up to the threshold below 0.5, indicating the application of the TLR4 and FLT3 models within this threshold range (Figure 5C). Moreover, the nomogram of the 2 variables of TLR4 and FLT3 was combined to predict the probability of PI occurrence (Figure 5D). In the ROC curve of the combined model of FLT3 and TLR4, an AUC = 1.00 implied that the joint model has perfect discriminatory power (Figure 5E). These analyses illustrated that TLR4 and FLT3 may play a crucial role in the pathogenesis of PI.

### 2.6. GSEA and GSVA of the Biomarkers

**Figure 6:**
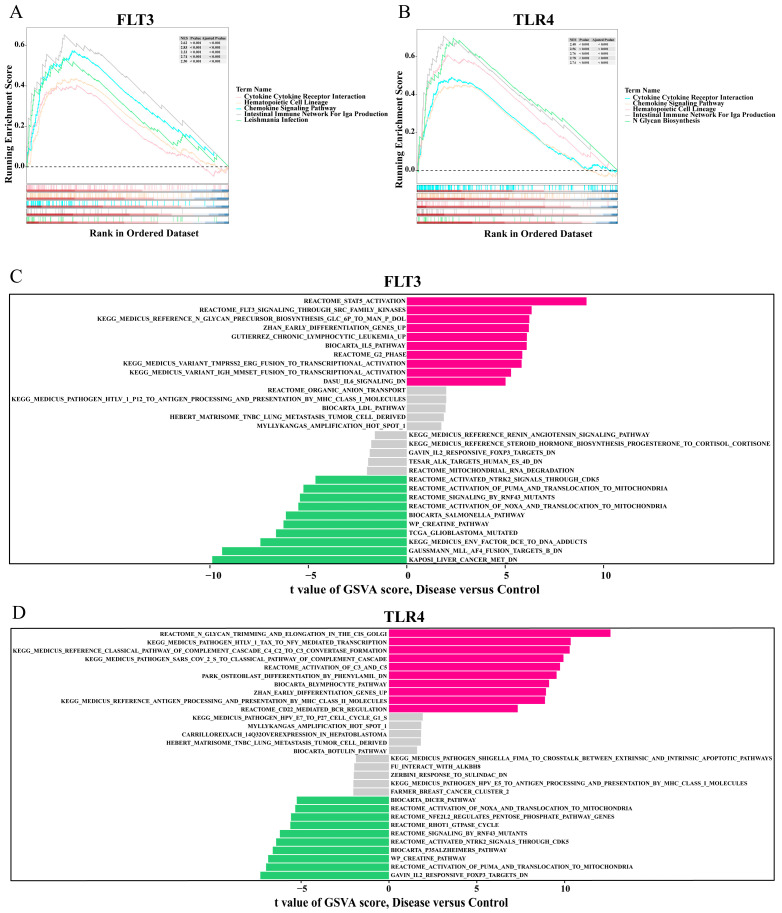
GSEA and GSVA of FLT3 and TLR4. (**A**,**B**) GSEA identifies pathways significantly associated with FLT3 and TLR4. (**C**,**D**) GSVA of FLT3 and TLR4. Pink strip: gene set expression was significantly up-regulated in the disease group; green stripe: the expression of the gene set was significantly up-regulated in the control group; grey strip: gene set with no significant difference in expression. The larger the absolute value of the t-score, the more significant the difference. GSEA, Gene Set Enrichment Analysis; GSVA, Gene Set Enrichment Analysis.

GSEA is particularly valuable for elucidating comprehensive and coherent changes in biological processes through gene set enrichment approaches. In the analysis of the training set, pathways significantly associated with FLT3 and TLR4 included cytokine–cytokine receptor interaction, the chemokine signaling pathway, and the intestinal immune network for IgA production (Figure 6A,B). Meanwhile, the GSVA demonstrated that in samples with high expression in the disease group of FLT3, there is notable enrichment in STAT5 activation and FLT3 signaling through Src family kinases. In samples with high expression in the control group of FLT3, it is predominantly enriched in Kaposi liver cancer Met and Gaussmann MLL-AF4 fusion targets B (Figure 6C). For samples with high expression in the disease group of TLR4, there is significant enrichment in N-glycan trimming and elongation in the cis-Golgi and Medicus pathogen HTLV-1 Tax to NFY mediated transcription. In samples with high expression in the control group of TLR4, it is mainly enriched in Gavin IL2 responsive FOXP3 targets and the activation of PUMA and translocation to mitochondria (Figure 6D). These findings contributed to a deeper understanding of the biological roles associated with these biomarkers in PI.

### 2.7. Immune Infiltration in PI

**Figure 7:**
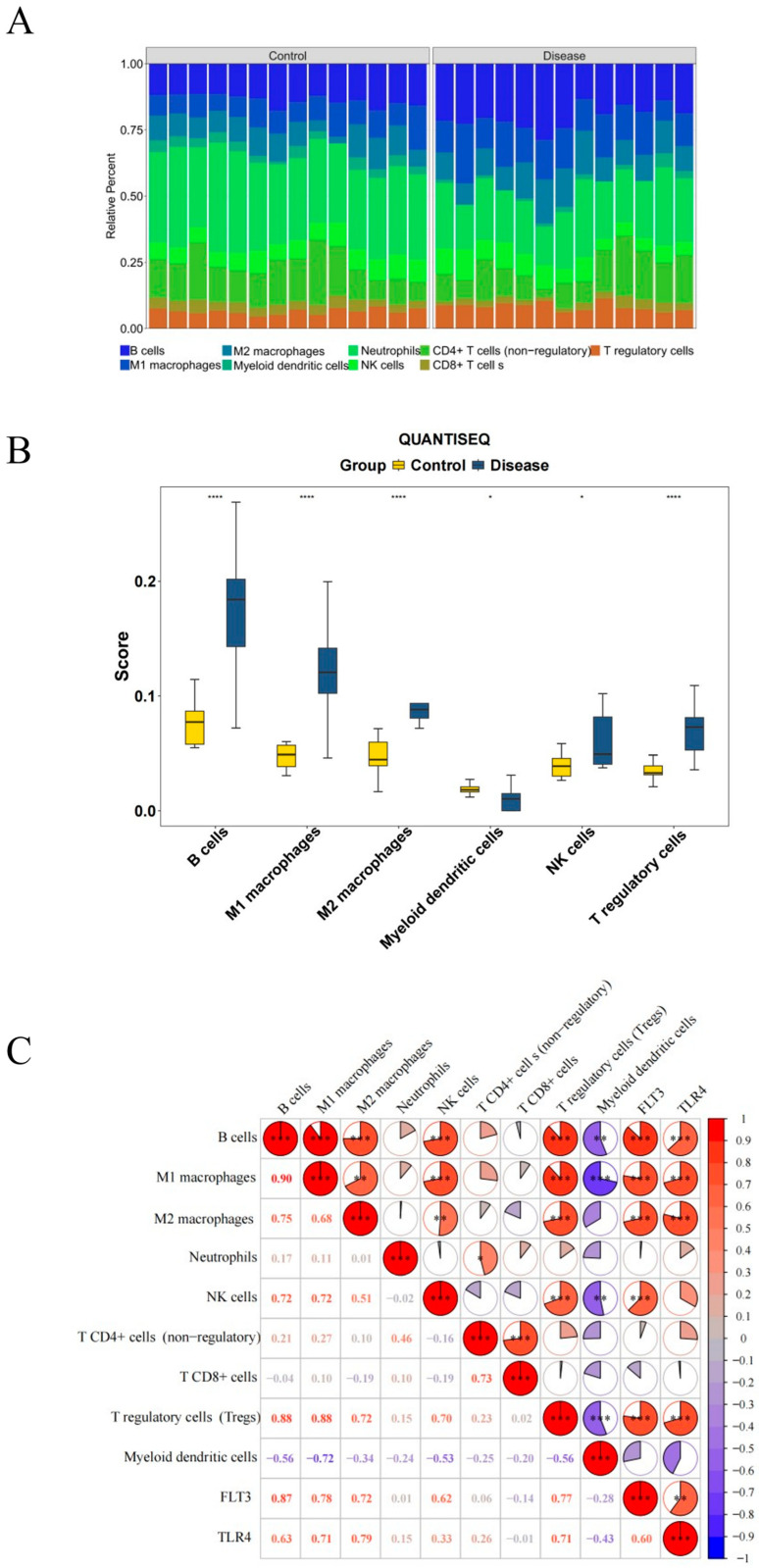
Immune infiltration in PI. (**A**) stacked bar plot for immune cell composition. (**B**) Box plot showing the distribution of immune cell infiltration levels between control group and disease group. (**C**) Correlation analysis between immune cells and between immune cells with FLT3 and TLR4. * *p* < 0.05; ** *p <* 0.01; *** *p <* 0.001; **** *p* < 0.0001.

To investigate the relationship between biomarkers and immune infiltrating cells, we analyzed the variations in the infiltration proportion of immune cells between the PI and control groups. Among them, there was a significant difference in six types of immune cells between the two groups, including B cells, M1 macrophages, M2 macrophages, myeloid dendritic cells, natural killer (NK) cells, and Tregs (*p* < 0.05) (Figure 7A,B). Among the immune cells, B cells demonstrated a positive connection with M1 macrophages (cor = 0.90, *p* < 0.05), and myeloid dendritic cells exhibited a pronounced negative correlation with M1 macrophages (cor = −0.72, *p* < 0.05). Moreover, FLT3 and TLR4 exhibited a positive correlation when combined with B cells, M1 macrophages, M2 macrophages, and Tregs (cor > 0.60, *p* < 0.05). However, TLR4 displayed a negative correlation with myeloid dendritic cells (cor = −0.43, *p* < 0.05) (Figure 7C). These discoveries highlighted specific immune cell types with significant differential infiltration and correlations with biomarkers, offering valuable insights into PI pathogenesis.

### 2.8. Network Construction for ‘Biomarkers-Oral Diseases’, ‘lncRNA-miRNA-mRNA’, and ‘miRNA-mRNA-TF’ Relationships

**Figure 8:**
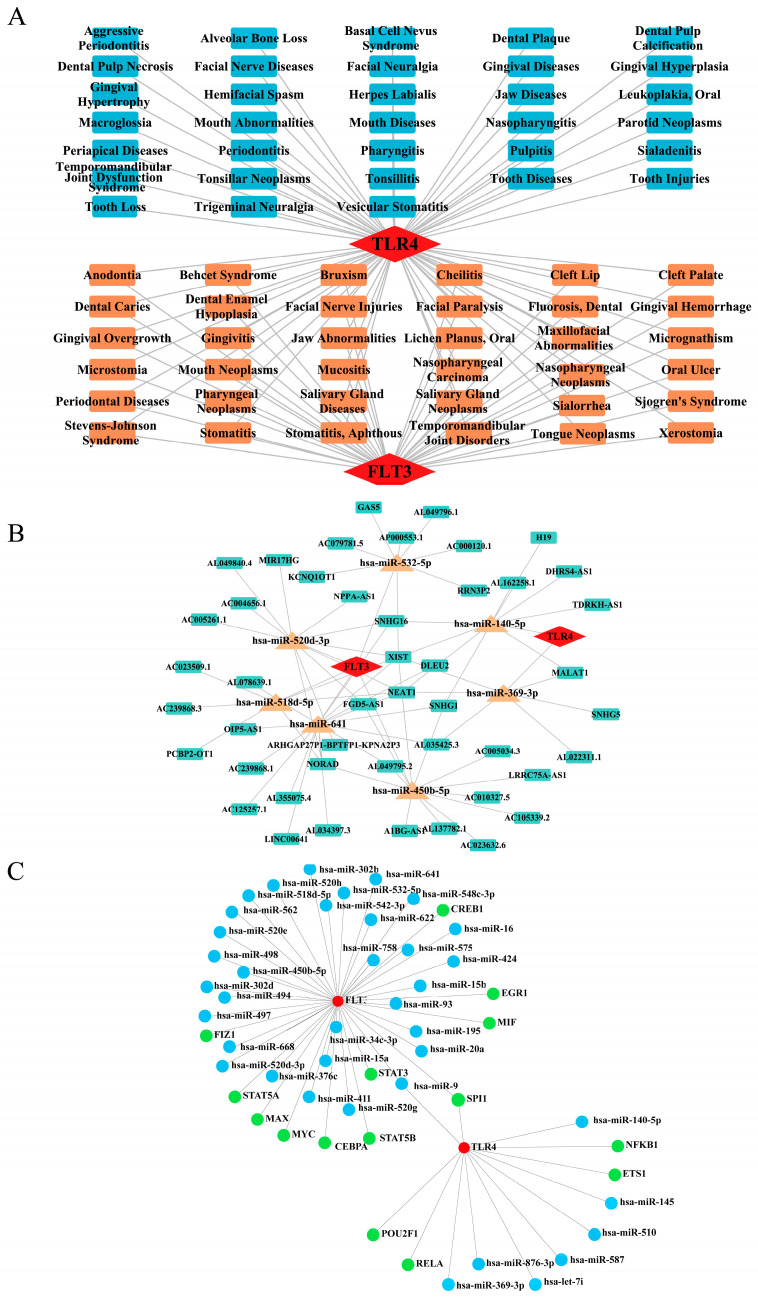
Network construction for ‘biomarkers-oral diseases’, ‘lncRNA-miRNA-mRNA’, and ‘miRNA-mRNA-TF’. (**A**) Association network between FLT3, TLR4, and oral diseases. Red squares: genes; blue squares: diseases associated only with the TLR4 gene; and orange squares: diseases associated with both genes. (**B**) ‘lncRNA-miRNA-mRNA’ regulatory network. Red squares represent genes; blue squares represent lncRNAs; yellow squares represent miRNAs. (**C**) ‘miRNA-mRNA-TF’ regulatory network. Red nodes represent genes; blue nodes represent miRNAs; and green nodes represent TF. TF, transcription factors.

In the “biomarkers-oral diseases” network, TLR4 was associated with 69 oral diseases, and FLT3 was associated with 35 oral diseases. Among them, gingival overgrowth, oral ulcer, and bruxism were all associated with both biomarkers, suggesting possible synergistic effects between TLR4 and FLT3 (Figure 8A). The “lncRNA-miRNA-mRNA” regulatory network revealed that 7 miRNAs and 70 lncRNAs interacted with the biomarkers, including relationships such as XIST-“hsa-miR-520d-3p”-FLT3, NORAD-“hsa-miR-518d-5p”-FLT3 and NEAT1-“hsa-miR-369-3p”-TLR4 (Figure 8B). Furthermore, 15 TFs were predicted to be biomarkers. The TFs for each biomarker were screened to construct a “miRNA-mRNA-TF” network. Noteworthy miRNAs and TFs that had strong connections with biomarkers encompassed “hsa-miR-9”-TLR4-SPI1, “hsa-miR-9”-TLR4-NFKB1, and “hsa-miR-16”-FLT3-STAT3 (Figure 8C). In summary, these network analyses strongly supported elucidating the regulatory mechanisms underlying PI.

### 2.9. TLR4 and FLT3 Function Varied Significantly Between the Different Subtypes

**Figure 9:**
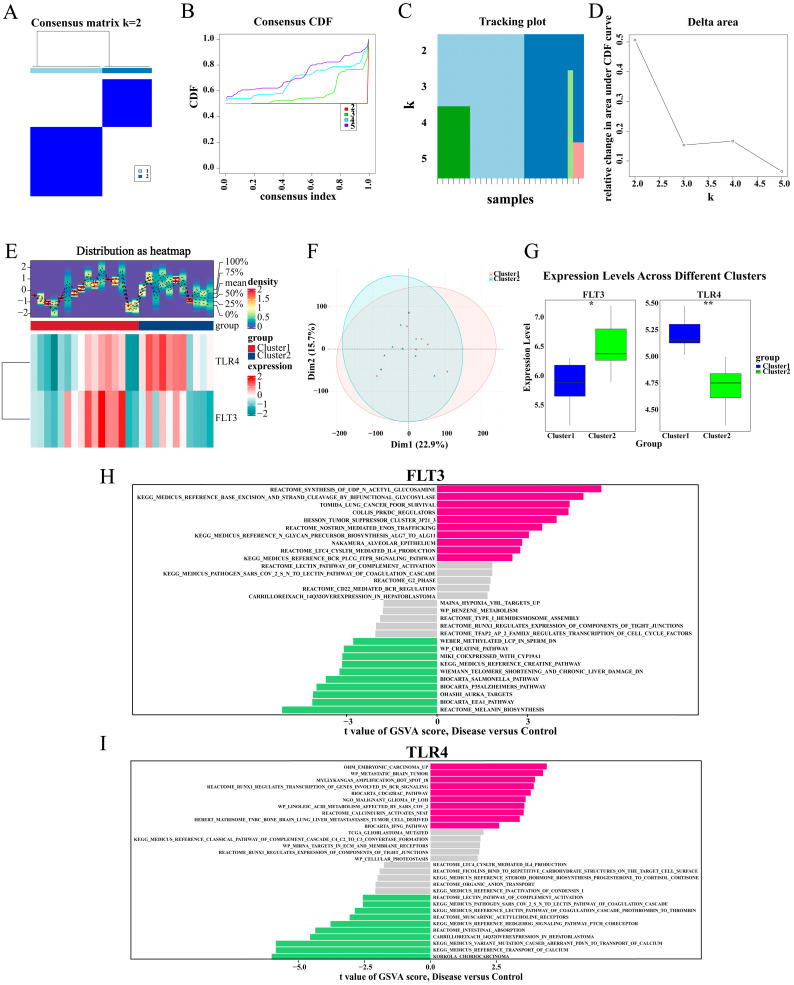
TLR4 and FLT3 functions varied significantly between the different subtypes. (**A**) Consensus Matrix, displaying the stability of the data under a cluster number of (k = 2). (**B**). Consensus CDF (cumulative distribution function), showing the CDF curves, which represent the CDF values under different cluster numbers (k = 2, 3, 4, 5). The smoother the curve, the more stable the clustering result. k = 2 may be an optimal clustering choice because it maximizes the consistency of samples within the same cluster. (**C**). Delta Area Plot, showing the relative change in the area under the CDF curve to assess the impact of different k values on clustering stability. The area change is largest at k = 2. (**D**). Tracking Plot, displaying the distribution of sample clustering under different cluster numbers k. At k = 2, the samples are divided into two distinct groups (blue and light blue). (**E**) Heatmap and density distribution of FLT3 and TLR4 gene expression in different groups. (**F**) PCA map of FLT3 and TLR4 gene expression. (**G**) Box plot of FLT3 and TLR4 gene expression between two subgroups. (**H**,**I**) GSVA of FLT3 and TLR4. Pink strip: gene set expression was significantly up-regulated in the disease group; green stripe: the expression of the gene set was significantly up-regulated in the control group; grey strip: gene set with no significant difference in expression. CDF, cumulative distribution function; GSVA, Gene Set Enrichment Analysis. * *p* < 0.05; ** *p* < 0.01.

The consensus clustering analysis was employed to determine the robust subpopulations within the data. The outcomes demonstrated that K = 2 was the optimal number of clusters for the data, as it exhibited good sample consistency and stability (Figure 9A–D). The expression levels of FLT3 and TLR4 in cluster1 and cluster2 were presented in the heatmap (Figure 9E). The samples from cluster1 and cluster2 were somewhat separated in the principal component analysis, signifying that the two groups differ in their gene expression profiles (Figure 9F). This separation further corroborated the differential expression of the genes FLT3 and TLR4 in the two cohorts. FLT3 expression was notably higher in cluster2 than in cluster1 (*p* < 0.05), while TLR4 exhibited the opposite trend and more significant differences (*p* < 0.01) (Figure 9G). These findings were of great importance as they yielded valuable revelations about the heterogeneity of the data and the potential differences in the underlying biological processes.

Figure 9H shows the GSVA results of different subtypes for the FLT3 gene level. The pathways upregulated in the disease group may be related to the synthesis of UDP-N-acetylglucosamine, base excision, and strand cleavage by bifunctional glycosylase. The pathways downregulated in the disease group may involve melanin biosynthesis and the EEA1 pathway. Figure 9I presents the differences in TLR4 gene-related pathways between the disease and control groups based on GSVA. Among them, embryonic carcinoma, metastatic brain tumor, and amplification hot spot 18 were significantly upregulated in the disease group, while choriocarcinoma and calcium transport were significantly downregulated in the disease group. These results demonstrated the potential mechanisms of FLT3 and TLR4 in the progression of PI and helped to reveal their functional differences among different subtypes.

### 2.10. Fedratinib and Ibudilast Might Represent Potential Drugs for Treating PI

**Figure 10:**
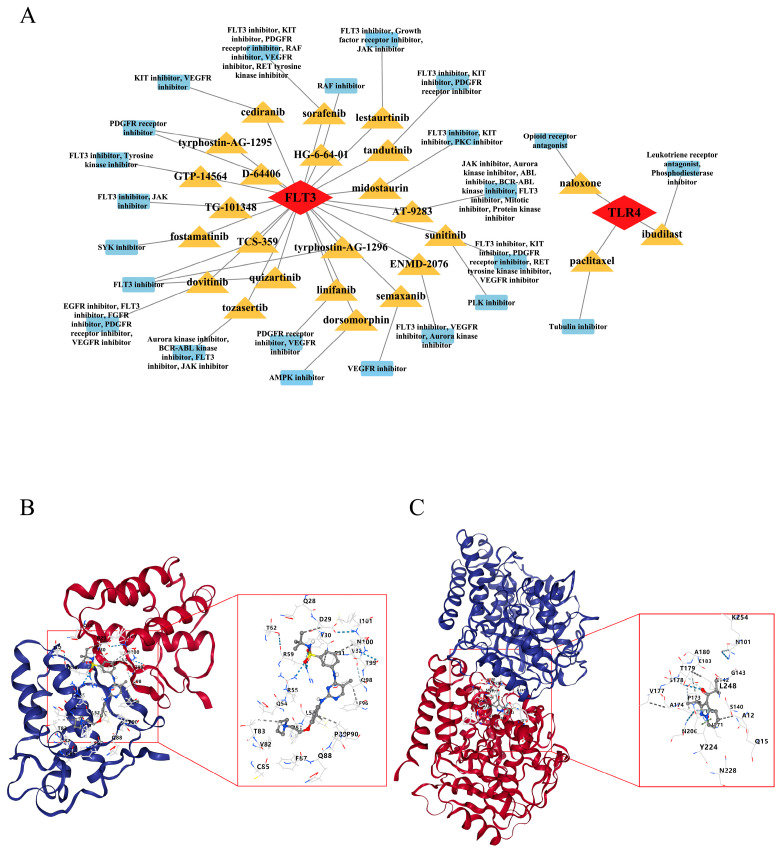
Drug prediction targeting FLT3 and TLR4 and molecular docking of candidate drugs. (**A**) Drugs targeting FLT3 and TLR4 and their mechanisms of action. The red squares represent the FLT3 and TLR4 genes, the orange squares indicate specific drug names, and the blue squares show the mechanisms of action of these drugs. (**B**) Docking analysis of FLT3 with Fedratinib. The red and blue helices represent different domains or regions of the FLT3 protein; the gray stick model represents Fedratinib; blue dashed lines indicate hydrogen bond interactions; and gray dashed lines indicate hydrophobic interactions. (**C**) Molecular docking analysis of TLR4 with Ibudilast. The red and blue helices represent different domains or regions of the TLR4 protein; the gray stick model represents Ibudilast; blue dashed lines indicate hydrogen bond interactions; and gray dashed lines indicate hydrophobic interactions.

In the “biomarker-drug-mechanism” network, there were 22 drugs associated with FLT3 and 3 drugs related to TLR4. Notably, FLT3 was predicted to be linked to fedratinib (TG-101348), which was related to the FLT3 inhibitor and JAK inhibitor. TLR4 was expected to have a connection with ibudilast, which was related to the leukotriene receptor antagonist and phosphodiesterase inhibitor (Figure 10A). Based on the above results, we selected fedratinib and ibudilast as the most suitable candidates from among the drugs with high enrichment scores. Subsequently, cb-Dock was employed to conduct molecular docking validation. Both biomarkers showed good binding properties with the drugs. Specifically, FLT3 showed a binding vina score with fedratinib of −8.3 kJ/mol, where the residues, such as D29, Y30, and R59, formed hydrogen bonds with fedratinib (Figure 10B). TLR4 exhibited a binding vina score with ibudilast of −7.7 kJ/mol, where A180, T179, and E183 residues were involved in hydrogen bonding (Figure 10C). The Vina scores of all drugs related to TLR4 and FLT3 are provided in Supplementary Tables S1 and S2. These findings suggested that these drugs may be potential therapeutic agents for treating PI.

### 2.11. The Construction of an Inflammatory State Model of HGFs and Detection of Key Biomarkers

**Figure 11:**
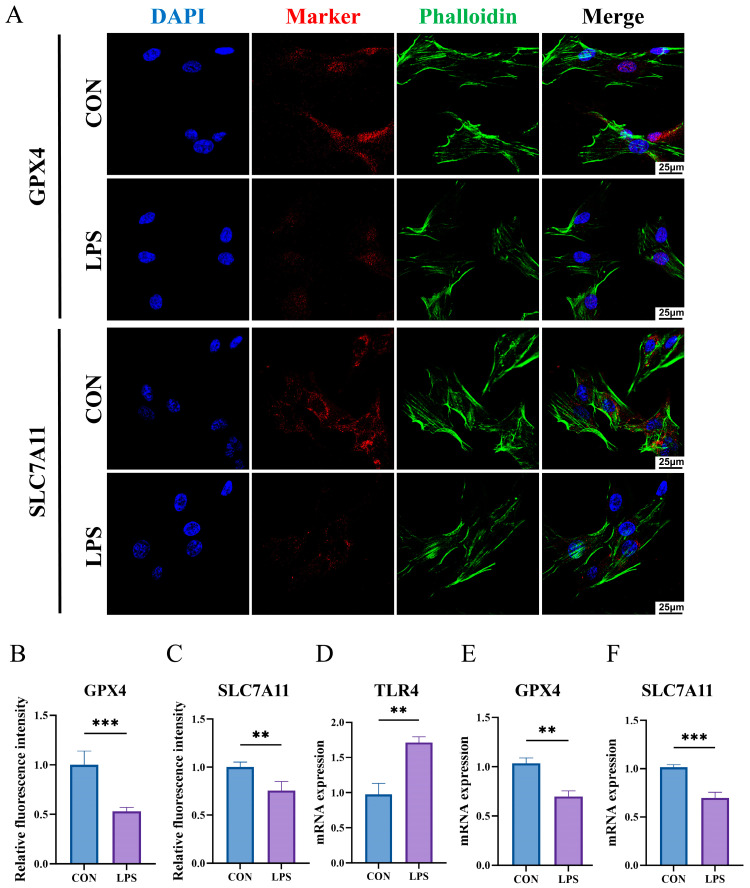
The construction of the in vitro PI model and detection of key biomarkers. (**A**) Immunofluorescence staining of GPX4 and SLC7A11 in HGFs treated with LPS (1 μg/mL), (*n* ≥ 3). (**B**) Relative fluorescence intensity of GPX4. (**C**) Relative fluorescence intensity of SLC7A11. (**D**–**F**) mRNA expression level of GPX4, SLC7A11, and TLR4. ** *p* < 0.01; *** *p* < 0.001.

We constructed an inflammatory state model of HGFs in vitro. Figure 11A shows the expression of GPX4 and SLC7A11 as analyzed by immunofluorescence staining. Figure 11B,C display the relative fluorescence intensity of GPX4 and SLC7A11, respectively. The results indicate that, after LPS treatment, the expression of both proteins significantly decreased (*p* < 0.01), suggesting the occurrence of ferroptosis. Similarly, the mRNA expression levels of GPX4 and SLC7A11 were also significantly reduced (*p* < 0.01) (Figure 11D,E). These results collectively suggest that ferroptosis may play a key role in the damage and inflammatory response of gingival fibroblasts. Importantly, the RT-qPCR results showed that TLR4 mRNA expression was upregulated in HGFs under inflammatory conditions, which is consistent with our bioinformatics analysis (Figure 11F).

## 3. Discussion

PI is a chronic inflammatory disease affecting the soft and hard tissues around the dental implant. Clinically, once PI occurs, it is often irreversible and carries a high risk of recurrence [30]. If adequate treatment interventions are lacking, the progression of PI tends to follow a non-linear, accelerated course [31]. Ferroptosis is a regulated cell death process triggered by iron accumulation and lipid peroxidation, both driven by ROS. Ozawa, Ryotaro, et al. developed a novel redox injectable gel containing nitroxide radicals as a ROS scavenger. This gel effectively reduced oxidative damage, prevented bone resorption, and mitigated loss of bone density in a rat model of PI [32]. Therefore, PI due to bacterial infection and inflammatory response can lead to the excessive production of ROS, inducing ferroptosis, which aggravates local tissue damage and bone resorption. Moreover, the release of various damage-associated molecular patterns (DAMPs) during ferroptosis can further activate inflammatory responses, creating a vicious cycle that exacerbates PI. Uncontrolled ferroptosis may accelerate bone loss and implant failure, thereby affecting treatment outcomes and reducing the long-term survival rate of implants. Ferroptosis may play a key role in PI, but its related mechanisms are unclear.

Here, we adopted comprehensive bioinformatics analyses, including differential expression analysis, enrichment analysis, protein–protein interaction networks, machine learning model construction, and drug molecule docking validation, to identify key regulatory genes in PI and explore potential therapeutic drugs. Our study identified 643 DEGs, with 374 upregulated and 269 downregulated, offering insights into the genetic regulation and pathways involved in PI. Notably, 13 PI-Ferr-DEGs were identified, including TLR4 and FLT3, which are considered potential regulatory genes that provide targets for therapeutic intervention. Machine learning models highlighted TLR4 and FLT3 as candidate biomarkers with diagnostic potential. Immune cell infiltration analysis revealed an increase in macrophages and NK cells in PI, indicating changes in the immune microenvironment during the progression of PI. Drug prediction and molecular docking identified fedratinib and ibudilast as potential therapeutics for PI. This study provides a solid framework for understanding PI’s molecular mechanisms and improving diagnostic and therapeutic strategies.

TLR4 and FLT3 are expected to become emerging diagnostic biomarkers and therapeutic targets for PI. TLR4 is a pattern recognition receptor predominantly situated on the cell membrane, where it plays a crucial role in the innate immune system by recognizing DAMPs [33]. During the process of ferroptosis, cells release multiple DAMPs, such as high mobility group box 1 (HMGB1) and heat shock proteins (HSPs) [34]. These DAMPs can activate the TLR4 receptor, inducing downstream inflammatory responses, such as the induction of the nuclear factor κB (NF-κB) pathway, leading to the release of pro-inflammatory cytokines like tumor necrosis factor-α (TNF-α), interleukin-1β (IL-1β), and interleukin-6 (IL-6) [35,36]. This inflammatory response can exacerbate the cellular oxidative stress state, further enhancing the generation of ROS. Excessive ROS is one of the main inducers of ferroptosis, ultimately further promoting the development of ferroptosis. In peri-implantitis, persistent inflammatory responses may lead to bone resorption, which is associated with the activation of osteoclasts. The activation of TLR4 can promote the differentiation of osteoclast precursors, thereby aggravating bone loss [37]. A study has found that the preventive application of melatonin can reduce the protein expression level of TLR4, thereby reducing the incidence of PI, suggesting its key role in this pathological state [38]. In this study, we found that TLR4 was significantly upregulated in HGFs under inflammatory conditions, while FLT3 was almost not expressed in HGFs. This suggests that TLR4 may play a key role in the pathological process of HGFs under inflammatory conditions, and the specific mechanisms still require further experimental investigation.

FLT3 is a receptor tyrosine kinase primarily involved in developing hematopoietic cells and regulating immune responses [39]. RNAi against FLT3 can rescue cells from ferroptosis by limiting lipid peroxidation and inactivating p22phox [40]. Kang Y et al. also demonstrated the role of FLT3 inhibition in suppressing lipid peroxidation and ROS production, ultimately leading to the inhibition of ferroptosis [41]. The role of FLT3 in PI remains to be explored, though it has been somewhat investigated in inflammatory diseases [42]. For instance, the inhibition of FLT3 signal transduction by lestaurtinib can alleviate LPS-induced acute pulmonary inflammation and injury [43]. The GSVA results of this study indicate that in the PI group with high expression of FLT3, the signal transducer, and the activator of transcription 5 (STAT5) are activated (Figure 6C). When the FLT3 receptor is activated and autophosphorylated, it can further activate downstream Janus kinase (JAK), leading to the phosphorylation of STAT5. Subsequently, STAT5 activates and translocates to the nucleus, regulating gene expression related to inflammation, cell proliferation, survival, and other processes. The JAK-STAT signaling pathway can affect intracellular ROS production by regulating oxidative stress levels [44]. In an inflammatory environment, the upregulation of FLT3 expression and the activation of JAK-STAT5 may increase the generation of ROS, and excessive ROS are a core driving factor for ferroptosis. ROS induces the accumulation of lipid peroxides, which leads to the occurrence of ferroptosis. A deeper understanding of the relationship between the FLT3-JAK-STAT5 pathway and ferroptosis is essential for exploring potential therapeutic targets and prevention strategies for PI.

Immune dysregulation, particularly the imbalance between pro-inflammatory and anti-inflammatory responses, is a significant cause of disease exacerbation. Therefore, regulating the immune system while suppressing excessive inflammatory reactions is crucial for restoring tissue homeostasis and treating PI. Immune infiltration analysis indicated that, compared with the healthy group, the proportion of B cells, M1 macrophages, M2 macrophages, NK cells, and T regulatory cells was higher in the PI group. In contrast, the proportion of myeloid dendritic cells was less, and all these differences were statistically significant (Figure 7A,B). The results of this study are similar to those of a recently published work that describes the distribution patterns of immune cells and blood vessels in PI lesion tissues from a topological perspective [45]. We all found more macrophages and NK cells in the PI lesion tissues. Macrophage polarization refers to differentiating macrophages into M1 or M2 types in an inflammatory environment to meet physiological needs. The M1 type resists pathogens through pro-inflammatory cytokines (such as IL-6 and TNF-α) and phagocytosis. In contrast, the M2 type supports tissue repair and suppresses excessive inflammation through anti-inflammatory factors (such as IL-10 and TGF-β) and tissue remodeling [46]. This study found that, compared to the healthy group, the proportion of both M1 and M2 macrophages was higher in the PI group, with a higher proportion of M1 macrophages, suggesting that PI is in a state of persistent inflammation, leading to tissue damage and disease progression. Furthermore, correlation analysis indicated that TLR4 and FLT3 are positively correlated with both types of cells (Figure 7C). In PI, the activation of TLR4 and FLT3 may lead to more macrophages polarizing towards the M1 phenotype, enhancing pro-inflammatory responses and increasing the occurrence of ferroptosis. Although M2-type cells also increase, they are not sufficient to effectively inhibit this process. This dynamic balance affects the severity of inflammation and the efficiency of tissue repair. Studying these interactions may provide new insights into modulating inflammatory responses.

NK cells are innate immune cells that regulate immune responses and maintain immune balance by directly killing abnormal cells and releasing cytokines, such as interferon-γ (IFN-γ) [47]. Its role in pathological iron may be double-edged. On the one hand, a high proportion of NK cells in chronic inflammation can enhance the clearance of infections or mutated cells, aiding in the control of inflammation. On the other hand, the release of pro-inflammatory cytokines by NK cells may lead to a sustained inflammatory state, and, by influencing macrophages to polarize towards the M1 type and promoting oxidative stress in the inflammatory microenvironment, they may indirectly facilitate ferroptosis, ultimately exacerbating tissue damage. Therefore, further elucidating the role of NK cells in PI is important for controlling its inflammatory symptoms.

Ibudilast and Fedratinib are drugs targeting TLR4 and FLT3, respectively. There are no reports yet of their use in treating PI, making them noteworthy candidates for further development (Figure 10). In the prediction of potential TLR4-targeting drugs, although Paclitaxel and Naloxone received relatively high scores, they were excluded from further consideration due to reports of pro-inflammatory effects or adverse events [48,49,50,51]. Unlike the above, Ibudilast has demonstrated consistent anti-inflammatory activity across various disease models, and is therefore considered a promising candidate for PI therapy. Ibudilast is an orally administered capsule that acts as a phosphodiesterase-4 (PDE4) inhibitor, and it is already marketed in Japan [52]. PDE4 is a member of the phosphodiesterase (PDE) family and is widely present in various immune cells and airway smooth muscle [53]. Its primary function is to catalyze the degradation of cyclic adenosine monophosphate (cAMP). The upregulation of cAMP can inhibit the activation of NF-κB via the protein kinase A (PKA) pathway, thereby weakening or suppressing subsequent pro-inflammatory responses [54]. Therefore, as an inhibitor, Ibudilast can exert anti-inflammatory and immunomodulatory effects by increasing cAMP levels. Fedratinib is best known for its ability to inhibit JAK2 and is an oral medication used in the treatment of myelofibrosis [55]. In the screening of FLT3-targeted drugs, although Sorafenib, Quizartinib, and Linifanib showed higher Vina scores, they were excluded from further investigation due to their potential pro-inflammatory effects, safety concerns, and insufficient clinical data [56,57,58]. In contrast, Fedratinib, with its well-defined anti-inflammatory mechanism and supporting clinical data, is considered the most promising drug candidate. Fedratinib can attenuate the inflammatory response by regulating the JAK2/STAT3 signaling pathway in vitro [59]. A report indicates that Fedratinib can strongly inhibit the expression of inflammatory markers such as IL-1β and IL-6 in macrophages stimulated by LPS and IFN-γ [60]. Accordingly, the repurposing of these two drugs may provide a cost-effective option for developing new therapeutic strategies for PI.

The clinical heterogeneity in peri-implantitis definitions across datasets introduces a fundamental limitation that may influence the comparability and interpretation of biological findings. The inclusion criteria and clinical definitions of peri-implantitis varied across the three datasets used in this study (Supplementary Table S7). GSE106090 and GSE33774 both defined peri-implantitis as probing depth ≥ 5 mm with radiographic bone loss >3 mm at implants functioning for more than one year; however, GSE106090 used calibrated digital radiographs to quantify bone loss, while GSE33774 relied on clinical protocols without detailed measurement calibration. GSE223924 applied stricter criteria, requiring probing depths ≥ 6 mm and the presence of bleeding and/or suppuration on gentle probing, potentially indicating more severe or active disease. These differences may lead to variation in the biological phenotype of the peri-implantitis lesions represented in each dataset, such as chronic vs. acute inflammation, tissue destruction extent, and microbial environment. While batch correction and normalization were implemented to reduce technical variability, the underlying clinical heterogeneity remains a limitation.

Our study specifically explored the relationship between ferroptosis-related genes and PI, providing detailed evidence that builds on prior findings. Through a comprehensive approach that integrates bioinformatics and machine learning algorithms, we successfully identified TLR4 and FLT3 as biomarkers with substantial research potential. We performed functional enrichment analysis, immune infiltration assessments, and drug predictions, offering new perspectives for PI diagnosis and treatment. While previous studies have conducted bioinformatics and immune infiltration analyses on PI, their results often vary due to factors such as small sample sizes, differences in dental sampling methods, patient physical condition variations, and the severity of lesion sites [22,61,62]. These discrepancies highlight the need for deeper investigation into the pathological processes associated with PI. Although we have preliminarily confirmed that TLR4 plays a key role in HGFs under inflammatory conditions, the specific function of another key biomarker, FLT3, still needs further investigation. This study also has the following limitations: the identified biomarkers, signaling pathways, and potential therapeutic drugs still need validation through various cellular and animal experiments.

## 4. Materials and Methods

### 4.1. Data Preparation

Gene expression information of the GSE33774 dataset, GSE106090 dataset, and GSE223924 dataset related to peri-implantitis (PI) was downloaded using the Gene Expression Omnibus (GEO) database http://www.ncbi.nlm.nih.gov/geo/ (accessed on 1 August 2024). Among this information, there were gingival tissue samples from 7 PI patients (disease group) and 8 healthy controls (control group), which were retained in GSE33774 (platform: GPL6244), and gingival tissue samples from 6 PI patients and 6 healthy controls were retained from GSE106090 (platform: GPL21827) [63,64]. The combined dataset of GSE33774 and GSE106090 was used as the training set for gene identification via the Combat function of the “sva” package (v 3.46.0) [65]. The GSE223924 dataset (platform: GPL24676) comprised gingival tissue samples from 10 PI patients, and 10 healthy controls were used as a validation set [66]. The characteristics of the three datasets, including demographic information and inclusion criteria for peri-implantitis tissue samples, are summarized in Supplementary Tables S3–S7. The ferroptosis-related genes (FRGs) were retrieved from the FerrDb database http://www.zhounan.org/ferrdb/current/ (accessed on 1 August 2024). Through the merging, deduplication, and removal of non-human genes of the three gene sets, namely “Driver”, “Suppressor”, and “Marker”, a total of 267 FRGs were obtained [67].

### 4.2. Normalization of the Obtained Data

All data were normalized to identify genes with similar trend changes across groups. The boxplot and data density plots of GSE33774 and GSE106090 showed the gene expression level distribution and density distribution for multiple samples, respectively. Principal component analysis (PCA) revealed differences in the two datasets and between the control and disease groups, which were used to remove batch effects via the “FactoMineR” package (v 2.7) [68].

### 4.3. Differential Expression Analysis and Function Analysis

To determine differential expressed genes (DEGs) related to PI, differential expression analysis was analyzed between the disease and control groups in the training set via the “limma” package (v 3.58.1) [69]. The analysis performed thresholds of |log2 fold change (FC)| ≥ 1 and adj.*p* < 0.05. Using the “ggplot2” package (v 3.4.4) [70] and “pheatmap” package (v 1.0.12) [71], the volcano plot and the heatmap were subsequently generated to exhibit the top 10 (up/down) most significantly regulated DEGs and the expression of the DEGs in the samples. To discover the biological functions and signaling pathways of the DEGs in PI pathogenesis, the “ClusterProfiler” package (v 4.10.0) [72] was employed to execute Gene Ontology (GO) and Kyoto Encyclopedia of Genes and Genomes (KEGG) enrichment analyses (adj.*p* < 0.05). GO enrichment analysis is an analytical approach to functionally annotate genes, including three parts: the biological process (BP), cellular component (CC), and molecular function (MF). KEGG assesses the most prominent metabolic and signaling pathways involving these genes.

### 4.4. Identification, Function Analysis, Protein–Protein Interaction (PPI) Network, and Correlation Analysis of PI-Ferr-DEGs

The intersection of DEGs and FRGs was employed to gain PI-Ferr-DEGs. The differences in the expression of PI-Ferr-DEGs between disease and control groups were determined using the Wilcoxon test in the training set (*p* < 0.05), and box line plots and the heatmap were generated via the “ggplot2” package (v 3.4.4) and “ComplexHepheatmapglmnet” (v 4.1.4) [73]. Subsequently, the GO and KEGG pathway enrichment analyses were also performed on PI-Ferr-DEGs to explore the possible biological pathways and pathways involved (*p* < 0.05). Moreover, to understand the PPI network of the PI-Ferr-DEGs, these PI-Ferr-DEGs were then imported into the Search Tool for the Retrieval of Interacting Genes (STRING) database https://cn.string-db.org/ (accessed on 1 August 2024) to establish a PPI network using the Cytoscape software (v 3.10.2) [74] with a confidence score threshold = 0.4 and false discovery rate (FDR) ≤ 0.05, aiming to understand their interactions better. Moreover, CytoHubba is a plugin within Cytoscape (v 3.10.2). The key nodes in the PPI network were identified through the neighborhood connectivity conservation (NCC) algorithm of CytoHubba. Finally, to comprehend the correlation between PI-Ferr-DEGs, the Spearman correlation between PI-Ferr-DEGs was analyzed using the “psych” package (v 2.4.1) [75] based on all samples of the PI training set (with the threshold reference of |correlation coefficient (cor)| > 0.3 and *p* < 0.05).

### 4.5. Machine Learning Screening for Identifying Candidate Biomarkers

To determine feature genes associated with PI-Ferr-DEGs, LASSO, SVM-RFE, and Boruta were used in the training set. LASSO regression analysis was conducted on the associated genes using the “glmnet” package (v 4.1-8) [76]. In LASSO regression, the coefficient of each gene was progressively compressed. The compression rule was to gradually reduce some unimportant genes to zero while ensuring accuracy, and some important genes would remain as feature genes. SVM-RFE used the “e1071” package (v 1.7-1) [77] to iteratively remove the genes with the smallest feature scores and filter out the feature genes with a higher discriminative ability. Another machine learning method, Boruta analysis, used the “Boruta” package (v 8.0.0) [78] to rank genes in order of importance and filter the feature genes. Finally, the intersection of feature genes in the LASSO, SVM-RFE, and Boruta methods was taken to obtain candidate biomarkers.

### 4.6. Validation of Biomarkers by Expression Levels, Receiver Operating Characteristic (ROC), and Nomogram

The differences in the expression of candidate biomarkers between PI and control groups were determined with consistent expression differences using the Wilcoxon test in both the training set and the validation set, and box line plots were drawn (*p* < 0.05) via the “ggplot2” package (v 3.4.4), which were defined as biomarkers. Moreover, to investigate the diagnostic capacity of the biomarkers to discriminate the PI samples from the control samples, the results in both datasets were plotted as an ROC curve through the “pROC” package (v 1.18.4) [79], and biomarkers with area under curve (AUC) values greater than 0.7 in both datasets were selected for further screening.

A nomogram was established via the “rms” package (v 6.7-1) [80] to assess the predictive ability of biomarkers for PI, and each factor’s score was quantified in points, with the accumulative scores for all factors represented as total points. To further appraise the accuracy of gene expression levels in the prediction of PI occurrence, a calibration curve was constructed using the “rms” package (v 6.7-1). Subsequently, to evaluate the reliability of the nomogram, the package “rmda” (v 1.6) [81] was employed to conduct decision curve analysis (DCA), allowing us to estimate its clinical utility and net benefit.

### 4.7. Nomogram of Biomarkers’ Combination

A nomogram was established via the “rms” package (v 6.7-1) to combine biomarkers to predict the probability of PI occurrence. To assess the diagnostic performance of the biomarkers’ combination, a biomarkers-based combination model was built using logistic regression. The ROC curves were employed to assess the classification performance of the joint model.

### 4.8. Gene Set Enrichment Analysis (GSEA)

To further identify the possible biological activities of biomarkers connected with FRGs in PI, the Spearman correlation coefficients of each biomarker and all other genes were calculated in the training set using the “psych” package (v 2.4.1), and related pathways corresponding to each biomarker were obtained according to the correlation coefficients from the largest to the smallest. Then, GSEA was utilized to search for the potential functions of the biomarkers using the “clusterProfiler” package (v 4.10.1). FDR < 0.05, *p* < 0.05, and |normalized enrichment score (NES) |> 1 were regarded as a significant enrichment result. The analysis used the “c2.cp.all.v2022.1.hs.symbols.gmt” as the reference gene set from the Molecular Signature Database (MSigDB) https://www.gsea-msigdb.org/gsea/msigdb (accessed on 6 August 2024), and the top five most significantly enriched pathways were selected for display.

### 4.9. Gene Set Variation Analysis (GSVA)

To analyze the differential activation pathways between the samples of the disease and the control groups in the training set, the background gene set was fetched from the MSigDB database https://www.gsea-msigdb.org/gsea/msigdb (accessed on 6 August 2024) based on the biomarkers’ expression level. The package “GSVA” (v 1.50.0) [82] was utilized to carry out GSVA for all biomarkers from the samples of two groups. Then, according to the difference analysis via the “ggplot2” package (v 3.4.4) between the two groups (|t-scores| > 2 and *p* < 0.05), pathways with activation were selected to generate an enrichment map.

### 4.10. Immune Infiltration Analysis

Studying the distribution of immune cells can provide valuable insights into the pathogenesis of PI. To further understand the distinction in immune cells between the disease and control group, 10 immune cell infiltrations [83] were analyzed, and the relative percent of immune cells was obtained in the training set using the Quantification of Immunogenic cell death Subtypes by RNA-seq (QUANTISEQ) algorithm. Subsequently, the distinction of the differential immune cell abundance between the two groups was analyzed by the Wilcoxon test (*p* < 0.05). Moreover, the correlations between immune cells were utilized to explore it, along with those between biomarkers and immune cells, which were both determined via the “psych” package (v 2.4.1) [84] and Spearman’s correlation method.

### 4.11. Gene-Disease Network Analysis and Molecular Regulatory Network

To explore the association between biomarkers and diseases, diseases significantly related to biomarkers were analyzed using the CLUE database https://www.clue.io (accessed on 1 August 2024). To acquire a comprehensive and detailed insight into the molecular regulatory mechanisms of these biomarkers, additional analyses were conducted. The targeted miRNA relationship pairs predicted by the NetworkAnalyst database https://www.networkanalyst.ca/NetworkAnalyst/home.xhtml (accessed on 9 August 2024) were used to identify relevant microRNAs (miRNAs) based on biomarkers. Subsequently, key long non-coding RNAs (lncRNAs) targeting the miRNAs upstream were fetched from the miranda database http://www.microrna.org (accessed on 9 August 2024). Subsequently, transcription factors (TFs) associated with biomarkers were predicted using the NetworkAnalyst database. Finally, “lncRNA-miRNA-mRNA” and “miRNA-mRNA-TF” networks were generated using the Cytoscape software (v 3.10.2).

### 4.12. Comprehensive Analysis of Biomarkers Between Different Subtypes

To explore the different subtypes in PI, the biomarker expression was analyzed on all PI samples in the training set using the “ConsensusClusterPlus” package (v 1.62.0) [85]. The K value was determined based on the observed cumulative distribution function (CDF) and the gravel plot’s relative change in the AUC. And the corresponding consistency matrix diagram was shown according to the K value (pItem and pFeature = 0.8, distance = euclidean, clusterAlg = km). The subtypes with the most significant difference were selected for subsequent analysis. Subsequently, differences between the different clusters were appraised using PCA analysis. Finally, the heatmap and boxplots were used to show the expression of biomarkers in other clusters. Moreover, to investigate whether there were disparities in the potential biological functions involved between the different subtypes obtained previously, GSVA enrichment analysis was conducted on the different subtype samples, and the single-sample gene set enrichment analysis (ssGSEA) score of the pathway was computed.

### 4.13. Drug Prediction and Molecular Docking

For the biomarkers obtained above and to explore potential drugs that may be related to PI treatment, the Connectivity Map (CMAP) database https://www.broadinstitute.org/connectivity-map-cmap (accessed on 19 August 2024) was used to investigate drugs and mechanisms with potential targeted biomarkers. Molecular docking is a widely employed technique in drug design for predicting binding affinities between molecular components and proteins and determining binding sites. First, the molecular structure of predicted drugs was retrieved from the PubChem database https://pubchem.ncbi.nlm.nih.gov/ (accessed on 19 August 2024) and used as the ligand. The structure of the biomarkers’ protein was then obtained from the Protein Data Bank (PDB) database https://www.rcsb.org/ (accessed on 19 August 2024). Both ligand and protein structures were processed using the CB-Dock database https://cadd.labshare.cn/cb-dock/php/index.php (accessed on 20 April 2025) for molecular docking simulations.

### 4.14. Cell Culture

Human primary gingival fibroblasts (HGFs) were purchased from iCell Bioscience Inc. (Shanghai, China). HGFs were cultured in HGFs-specific medium (iCell, Shanghai, China) consisting of primary fibroblast basal medium, primary fibroblast culture supplement, 10% fetal bovine serum, and 1% penicillin-streptomycin, at 37 °C and 5% CO_2_. The HGFs from passages 3–5 were used in the following experiments. Cells were treated with 1 μg/mL Lipopolysaccharide (LPS) (InvivoGen, San Diego, CA, USA) for 12 h to construct an inflammatory state model of HGFs in vitro.

### 4.15. Reverse Transcription Quantitative Polymerase Chain Reaction (RT-qPCR)

Total RNA was extracted from the HGFs cultures. A NanoDrop Spectrophotometer (NanoDrop2000, Wilmington, DE, USA) was used to analyze the concentration and quality of the screened qualified RNA. RNA was reverse transcribed using a PrimeScript™ RT reagent kit (Takara Clontech, Kyoto, Japan). Expressions of specific genes were analyzed by RT-qPCR using Takara TB-Green Premix Ex Taq (Takara Clontech, Kyoto, Japan). The expression levels of Gapdh were used as a reference housekeeping gene. The analysis of relative mRNA expression levels was conducted using the 2^−ΔΔCt^ method. Primers used in this study are listed in Table 1.

### 4.16. Immunofluorescence Staining (IF)

HGFs were seeded onto coverslips in 24-well plates. The experiment was divided into two groups: the blank control group and the LPS group. The LPS group was treated with 1 μg/mL LPS for 24 h to construct an inflammatory state model of HGFs in vitro. The cells were washed three times with PBS and fixed in 4% paraformaldehyde for 12 min. After fixation, the cells were permeabilized with 0.5% Triton X-100 (Solarbio, Beijing, China) and incubated with blocking buffer (Beyotime, Shanghai, China) at room temperature for 30 min to prevent nonspecific binding. Subsequently, the samples were incubated overnight at 4 °C with primary antibodies against GPX4 (Zenbio, Sichuan, China, 1:100) and SLC7A11 (Proteintech, Hubei, China, 1:100), respectively. After washing three times with PBS, the samples were incubated with CoraLite594-conjugated Goat Anti-Rabbit IgG (Proteintech, Hubei, China, 1:300) and SF488 Phalloidin (Solarbio, Beijing, China, 1:200) for 1 h at room temperature, respectively. The cell nuclei were stained with DAPI (Beyotime, Shanghai, China) for 5 min. Fluorescence images were captured using confocal scanning microscopy and were quantified with ImageJ software (version 1.8.0).

### 4.17. Statistical Analysis

The data related to bioinformatics analysis were processed using R language software (version 4.3.2). Comparisons were performed using the Wilcoxon test. The mean and standard deviation (SD) of the in vitro cell experiments were calculated. Data were statistically analyzed using the two-tailed Student’s *t*-test. Graphs were generated using GraphPad Prism 9 software. *p* < 0.05 was considered statistically significant.

## 5. Conclusions

In this study, two key common biomarkers of TLR4 and FLT3 with potential diagnostic value were identified from PI data and the ferroptosis database by integrating various bioinformatics analysis algorithms. A comprehensive multidimensional analysis was conducted, focusing on the disease dataset and its relationship with these biomarkers. Additionally, immune infiltration analysis was performed, revealing the proportions of immune cells in the PI training dataset and their associations with the key biomarkers. Further, through drug prediction and molecular docking techniques, two promising potential therapeutic agents for PI were identified. Finally, we revealed that one of the biomarkers, TLR4, was differentially upregulated in HGFs under inflammatory conditions, providing a basis for further research. However, we acknowledge that these findings are primarily derived from computational analyses and public datasets, and further validation using clinical samples and in vitro experiments is necessary to confirm the diagnostic and therapeutic potential of the identified biomarkers. In summary, this study offers new insights into the diagnosis and potential treatment of peri-implantitis and lays a foundation for future experimental and translational research.

## Figures and Tables

**Table 1:** Primer sequences used for RT-qPCR.

Gene	Acc. No	Primer Sequence(5′-3′)
TLR4	NM_003266.4	F:AGACCTGTCCCTGAACCCTAT R:CGATGGACTTCTAAACCAGCCA
GPX4	NM_001039847.3	F:GAGGCAAGACCGAAGTAAACTAC R:CCGAACTGGTTACACGGGAA
SLC7A11	NM_014331.4	F:TCTCCAAAGGAGGTTACCTGC R: AGACTCCCCTCAGTAAAGTGAC
GAPDH	NM_001256799.3	F: GGAGCGAGATCCCTCCAAAAT R: GGCTGTTGTCATACTTCTCATGG

## Data Availability

Datasets are freely available at http://www.ncbi.nlm.nih.gov/geo/ (accessed on 1 August 2024). All data originate from public sources.

## Supplementary Materials

The following supporting information can be downloaded at: https://www.mdpi.com/article/10.3390/ijms26094306/s1.
