# Supplementary material for: Integration of Bioinformatics and Machine Learning Strategies Identifies Ferroptosis and Immune Infiltration Signatures in Peri-Implantitis
Source: Int J Mol Sci. 2025 May 1;26(9):4306. doi: 10.3390/ijms26094306 (PMC12072437; doi:10.3390/ijms26094306)
Supplement: Supplementary file 1 [file ijms-26-04306-s001.zip › Supplemental Figure S1.pdf]

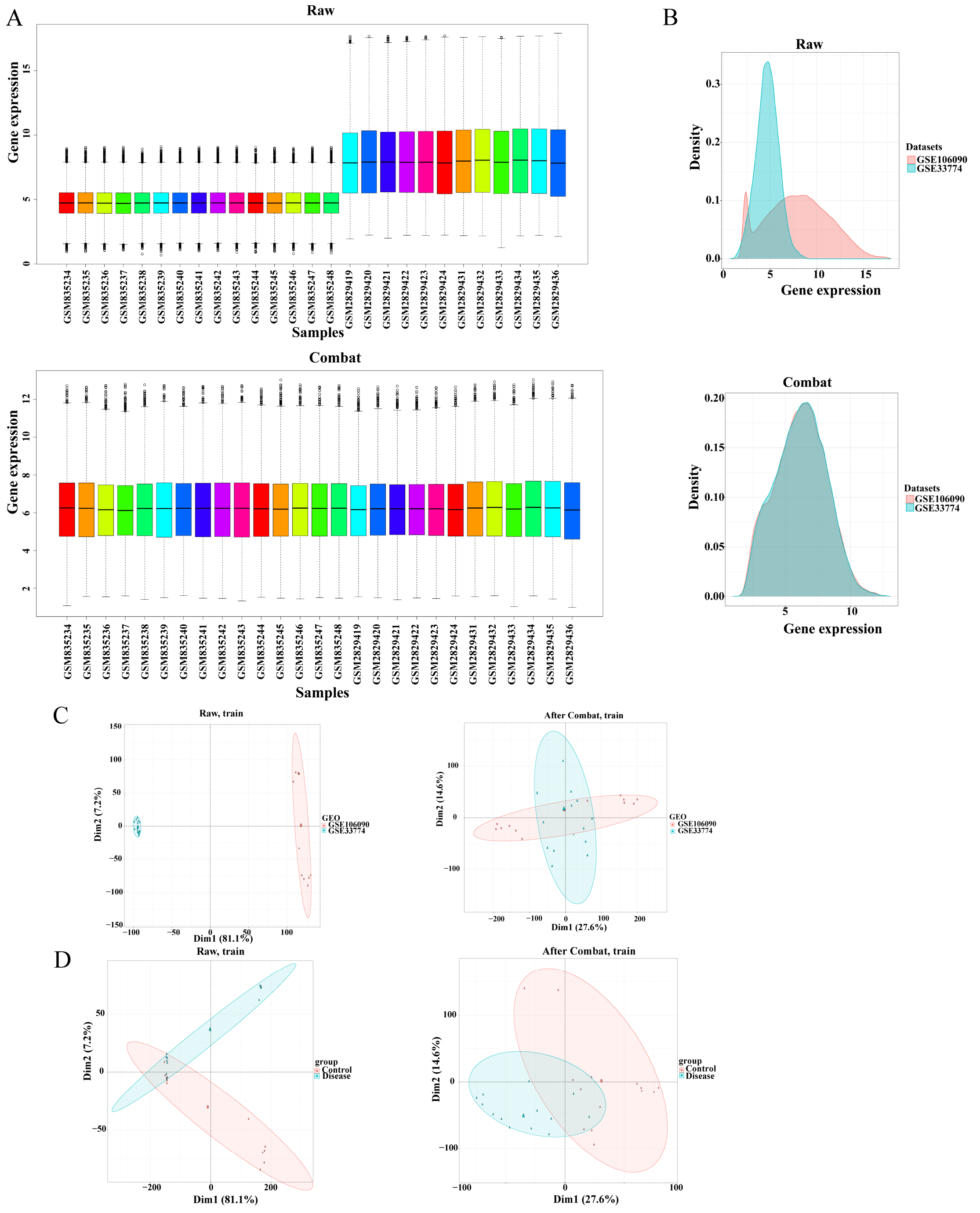

Supplemental Figure S1. Dataset normalization processing.

(A) The box plots of the original training set and the normalized training set. (B) The data density maps of the original training set and the normalized training set. (C) PCA maps of the original training set and the normalized training set. (D) Original and normalized PCA maps based on disease grouping.
