# Supplementary material for: Integration of Bioinformatics and Machine Learning Strategies Identifies Ferroptosis and Immune Infiltration Signatures in Peri-Implantitis
Source: Int J Mol Sci. 2025 May 1;26(9):4306. doi: 10.3390/ijms26094306 (PMC12072437; doi:10.3390/ijms26094306)
Supplement: Supplementary file 1 [file ijms-26-04306-s001.zip › Supplementary Tables.pdf]

Supplementary Table S1. Vina Scores of FLT3-Related Drugs

Name	Vina
sorafenib	-8.5
quizartinib	-8.5
linifanib	-8.5
dorsomorphin	-8.4
TG-101348 (fedratinib)	-8.3
tandutinib	-8.3
tozasertib	-8.2
lestaurtinib	-8.2
midostaurin	-8.1
dovitinib	-7.9
cediranib	-7.8
AT-9283	-7.7
D-64406	-7.6
fostamatinib	-7.6
ENMD-2076	-7.5
sunitinib	-7.4
TCS-359	-7.3
semaxanib	-7.2
tyrphostin-AG-1295	-7.2
GTP-14564	-7.1
tyrphostin-AG-1296	-7

Supplementary Table S2. Vina Scores of TLR4-Related Drugs

Name	Vina
paclitaxel	-12.5
naloxone	-8.1
ibudilast	-7.7

Supplementary Table S3. Characteristics of datasets included in this study

Dataset	Number of Samples	Disease Status	Sample Source	Demographic Info Available	Platform	PMID
GSE33774	Healthy: 8<br>PI: 7	Healthy/PI	Gingival tissue	Yes	GPL6244	**22967131**
GSE106090	Healthy: 6<br>PI: 6	Healthy/PI	Gingival tissue	Yes	GPL21827	**31853997**
GSE223924	Healthy: 10<br>PI: 10	Healthy/PI	Gingival tissue	Yes	GPL24676	**37789641**

Supplementary Table S4. Demographic information of GSE106090

	Healthy individuals (H)	Peri-implantitis patients (I)

n	6	6
Gender (female / male)	2/4	2/4
Age (years, Mean ± SD)	33.2 ± 8.0	55.7 ± 13.0
Probing Depth (mm, Mean ± SD)	1.8 ± 0.3	6.7 ± 0.8

Supplementary Table S5. Demographic information of GSE33774

	Healthy individuals	Peri-implantitis patients
n	8	7
Gender (female / male)	2/6	2/5
Median age (years)	32	57
Age range	14-78	38-71
Probing Depth (mm)	＜3mm	≥5mm

Supplementary Table S6. Demographic information of GSE223924

	Healthy individuals (H)	Peri-implantitis patients (I)
n	10	10
Gender (female / male)	5/5	5/5
Age (years, Mean ± SD)	65.9 ± 7.37	61.4 ± 6.09
Probing Depth (mm, Mean ± SD)	2.07 ± 0.21	6.03 ± 0.39

Supplementary Table S7. The inclusion criteria and clinical definitions of peri-implantitis

Dataset	Definition of Peri-implantitis	PD	Radiographic Bone Loss	Suppuration/Bleeding	Implant Function Time
GSE106090	PD >5 mm + BOP (+) + bone loss >3 mm	>5 mm	>3 mm (digital X-ray, calibrated by implant length)	BOP (+), suppuration not specified	>1 year
GSE33774	PD ≥5 mm + bone loss >3 mm	≥5 mm	>3 mm (not calibrated)	Not specified	>1 year
GSE223924	PD ≥6 mm + bone loss ≥3 mm + bleeding/suppuration	≥6 mm	≥3 mm apical to implant top	Bleeding and/or suppuration required	Not specified

PD: Probing Depth, BOP:Bleeding On Probing.
